# Real-world experience of diagnosis, disability, and daily management in parents of children with different genetic developmental and epileptic encephalopathies: a qualitative study

**DOI:** 10.1080/07853890.2024.2446702

**Published:** 2024-12-28

**Authors:** Cristina Garcia-Bravo, Domingo Palacios-Ceña, Ángel Aledo-Serrano, Javier Güeita-Rodríguez, Juan Francisco Velarde-García, Juan Nicolas Cuenca-Zaldivar, Romain Marconnot, María Cristina Alonso-Blanco, Jorge Pérez-Corrales, Carmen Jimenez-Antona

**Affiliations:** a Research Group of Humanities and Qualitative Research in Health Science of Universidad Rey Juan Carlos (Hum&QRinHS) & Research Group in Evaluation and Assessment of Capacity, Functionality and Disability of Universidad Rey Juan Carlos (TO+IDI), Department of Physical Therapy, Occupational Therapy, Physical Medicine and Rehabilitation, Universidad Rey Juan Carlos, Alcorcón, Spain; b Research Group of Humanities and Qualitative Research in Health Science of Universidad Rey Juan Carlos (Hum&QRinHS), Department of Physical Therapy, Occupational Therapy, Physical Medicine and Rehabilitation, Universidad Rey Juan Carlos, Alcorcón, Spain; c Epilepsy and Neurogenetics Program, Vithas Madrid La Milagrosa University Hospital, Vithas Hospital Group, Madrid, Spain; d Department of Nursing, Red Cross Nursing College, Madrid, Spain; e Department of Nursing and Physiotherapy, Universidad de Alcalá, Madrid, Spain

**Keywords:** Caregivers, disability, epilepsy, neurodevelopmental disorders, parents, qualitative research

## Abstract

**Purpose:**

This study describes the experience of parents of children with developmental and epileptic encephalopathies (DEE) and how the disease impacts their daily lives.

**Materials and methods:**

A descriptive qualitative study was conducted using purposeful sampling. Twenty-one parents of children with DEEs caused by SCN1A, KCNQ2, CDKL5, PCDH19, and GNAO1 variants were included. Data collection was based on in-depth interviews and researchers’ field notes. An inductive thematic analysis was performed.

**Results:**

Five themes emerged: (a) the diagnostic process, which describes the path from the time parents recognize the first symptoms until diagnostic confirmation is obtained; (b) the relationship with health professionals during the search for a diagnosis, which describes how the entire process is conditioned by the relationships established; (c) the world of disability, revealing how the disease and disability impact the life of the parents; (d) living day to day, the parents continuously change their plans in anticipation of the onset of a seizure; (e) the disease progression, a cause of great concern in the parents.

**Conclusions:**

Our results show the need to develop recovery programs that integrate health and social interventions to support parents of children with DEE in the process of diagnosis and disease management.

## Introduction

Developmental and epileptic and encephalopathies (DEEs) are a heterogeneous group of disorders characterized by early onset, often severe seizures, and electroencephalogram abnormalities, with developmental impairment that tends to worsen, at least partially, due to the epilepsy [1]. These disorders are characterized by seizures that are resistant to therapy, in addition to other neurological symptoms such as global motor delay, intellectual disability and communication disorders; and non-neurological symptoms such as orthopedic, digestive and growth problems, among others [2].

Currently, there are few targeted therapies for specific genetic phenotypes of DEE that improve seizure control, developmental problems, and comorbidities [3]. The most frequent genes causing early-onset DEE include CDKL5, SCN1A, KCNQ2, PCDH19, GNAO1 and SCN2A/SCN8A [4].

The symptoms of DEE are highly variable and heterogeneous, which makes early diagnosis difficult and causes diagnostic and therapeutic delays [5–8]. In addition, the role of professionals is fundamental for confirming the diagnosis of DEE, to avoid a negative impact on parents when they learn the news [5,9]. Moreover, the care of patients with DEE and their symptoms requires a change in parental and family roles, which may have social and occupational repercussions [10]. In addition, the lifestyle of the entire family must change in anticipation of an oncoming seizure [11], with a particularly negative impact on the parents of children with a DEE [12].

Previous studies [5,13] highlight the need to develop studies focused on the impact that DEEs have on the family, caregivers, and siblings. They also point out the importance of knowing the overall situation of families and the impact on their daily, social, and work activities.

The present study was conducted in Spain. Spain currently has a national health system with two levels of care: primary care (PC) and specialized care (SC) [14]. Primary care is the gateway to the healthcare system and through the PC physician and/or pediatrician, children with DEE are referred to the other specialties in hospitals (SC). In Spain, there is also a private health sector, although it is a minority compared to the public sector. In Spain there are national and international reference centers for the care of patients with rare and complex epilepsies, some of which belong to the European EpiCARE network (https://epi-care.eu/). However, the distribution of these centers does not cover the entire national territory. The effort to demand specific care for children with DEE and resources for such care lies with the associations of parents of children with DEE and the societies of medical specialists [15]. In the case of rare disease (RD) and DEE in Spain, many difficulties exist related to early diagnosis and treatment by the public health system, leading many parents of children with DEE to seek alternatives in the private sector [16].

The experience of having children diagnosed with DEE is highly individual, thus, qualitative research can provide information about the experience of patients and families and the impact of the disease on their lives [17]. The aim of this study was to describe the experience of parents with children with DEE caused by variants in SCN1A, KCNQ2, CDKL5, PCDH19, and GNAO1, some of the most frequent genes with common and differential characteristics, in relation to diagnosis, disease management and disability in their daily lives.

## Material and methods

The present research is part of a larger qualitative study that described the experiences and unmet health care needs of parents of children with DEEs [18,19]. The first part described how parents experienced symptom management, treatment acceptance and the therapeutic relationship [18]. Subsequently, the study explored how the disease was experienced by the partner, the siblings of the child with DEE, and the grandparents, facing challenges such as conflicts and changes in the couple’s relationship, and the search for a balance between care, family life and work [19]. In this part of the study, our results show the impact that DEEs have on the parents in relation to diagnosis, disease management and disability.

### Study design

A qualitative, descriptive study was conducted based on an interpretive framework [20]. This paradigm describes how people construct their own social reality and knowledge through reconstructions of their individual experiences [21]. The Standards for Reporting Qualitative Research (SRQR) [22] and the Consolidated Criteria for Reporting Qualitative Research (COREQ) were followed [23].

### Participants, sampling strategies and recruitment process

In the present study, a purposeful sampling approach was used [24], concretely the Maximum Variation Sampling Technique [24,25]. This technique is used when the researcher seeks to (a) select a purposive sample that represents a broader group of cases as closely as possible or (b) set up comparisons among different types of cases.

The inclusion criteria consisted of: (a) parents (mothers and fathers) of children diagnosed with DEEs with pathogenic or likely pathogenic variants in *SCN1A*, *KCNQ2*, *CDKL5*, *PCDH19* or *GNAO1*; (b) having children between the age of four and 10 (both included); (c) a genetic diagnosis of their child performed at least one year beforehand; (d) Parents residing in Spain. Exclusion criteria were parents of children with genetic variants other than those referred to in the inclusion criteria; parents of a child who was ill during the recruitment period; and parents of an affected child who has not presented epileptic seizures at any time.

In qualitative research, there is no formula for the prior calculation of the sample size [20,24]. Due to the unavailability of many cases of each variant, all available cases of each variant were included to obtain a greater richness of the data. Previous qualitative studies on the perspective of parents of children with DEE used a sample size of between 12 and 20 cases for each variant [7,26,27]. The following patient advocacy groups were contacted: Apoyo Dravet, Asociación KCNQ2 España, Asociación de afectados CDKL5, Asociación Epilepsia rosa PCDH19 and GNAO1 España. Potential participants were contacted through the directors of each patient association. Forty-three parents who met the inclusion criteria were contacted (eight from the SCN1A-Dravet association, nine from KCNQ2, nine from CDKL5, 11 from PCDH19, six from GNAO1). After confirming the inclusion criteria, only 21 participants were included: four parents from SCN1A, three from KCNQ2, four from CDKL5, five from PCDH19, and five from GNAO1. The main reason for not participating was the limited time available to the couples. This lack of time involved modifying childcare schedules, changing family routines, medical appointments, and work schedules.

### Data collection

Individual, semi-structured interviews were conducted from May to July 2021 [24]. Table 1. Open-ended follow-up questions were also used to obtain the detailed descriptions.

**Table 1.** Semi-structured interview guide.

Research areas	Questions
Disease	What is it like to live with a child who has developmental and epileptic encephalopathies (DEE)? What is most relevant to you? How do you manage the symptoms of the disease?
Diagnosis	What was the process like leading up to the diagnosis? What was the most relevant aspect of this process? How was the moment of receiving the diagnosis of the disease? What was the most relevant aspect?
Impact of the disease	How does it affect your daily life? What are your expectations with your child about the disease and its evolution? How does the disease affect your family life? What do you think about the impact or repercussion of the disease on some members of your family (grandparents, siblings, etc.)?
Expectations for the future	What are your expectations for the future? If you could go back in time, would you change anything? Why?

Thereafter, the researchers noted the key words and themes identified in the participants’ responses and used their answers to ask for them to clarify the content [24]. The interviews were conducted *via* a private video chat room using the Microsoft Teams platform (https://www.microsoft.com/es-es/microsoft-teams/log-in). Each participant received a private and personalized email with an invitation. All interviews were conducted by four researchers (CGB, CJA, JFVG, and JGR). All interviews (*n* = 21) were audio-recorded, recording a total of 1659 min of interviews (the average duration of each interview was 69.12 ± 37.9 min). Additionally, researcher field notes were used as a secondary source of information [24].

### Data analysis

An inductive thematic analysis was applied [20,28]. Verbatim transcripts were drafted for each of the interviews and researchers’ field notes. Thematic analysis consisted of identifying the most descriptive content to obtain meaningful units (codes), and subsequently reduce and identify the most common meaningful groups (categories) [20,28]. In this manner, groups of meaningful units were formed, i.e. similar points or content that enabled the emergence of the topics that described the study participants’ perspective. Thematic analysis was applied separately to interviews and field notes by CGB, CJA, JFVG, and JGR. Joint team meetings were held to combine the results of the analysis and discuss data collection and analysis procedures. In these team meetings the final themes were displayed, combined, integrated, and identified. In case of divergence of opinions, the identification of the theme was based on consensus among the members of the research team. No qualitative software was used for the data analysis.

### Rigor

The techniques performed and application procedures used to control trustworthiness are described in Table 2 [20,29]. In relation to member checking, in the present study this technique was carried out with the purpose of asking the participants to develop or clarify the information provided in the interviews (primary data) [30]. Also, for the material and methods section of this manuscript, the work of Martín-Sanz et al. [31] and Boritz et al. [32] were used as a guide.

**Table 2.** Trustworthiness criteria.

Criteria	Techniques performed
Credibility	Researcher triangulation: each interview was analyzed by two researchers. Subsequently, team meetings were held in which the analyses were compared, and themes were identified.
	Triangulation of data collection methods: semi-structured interviews were conducted, and the researchers made field notes.
	Member checking: this consisted of asking participants to confirm the data obtained during data collection. All participants were offered the opportunity to review the audio and/or video recordings to confirm their experience. None of the participants made any additional comments.
Transferability	In-depth descriptions of the study were made, detailing the characteristics of the investigators, participants, sampling strategies, and data collection and analysis procedures.
Dependability	Audit by an external researcher: an external researcher evaluated the study’s research protocol, focusing on aspects related to the methods applied and the study design.
Confirmability	Researcher triangulation, data collection and analysis triangulation. Researcher reflexivity was encouraged through reflective reporting and description of the rationale for the study.

### Ethics

This study adhered to the principles of the Declaration of Helsinki. Ethical approval was granted by the Universidad Rey Juan Carlos Research Ethics Committee (code: 0803202107121). All participants (mother or father) gave their written consent to participate in the study. No children were included in the study.

## Results

Twenty-one parents of children with DEE were recruited (four male-19.04%). The mean age was 41.9 years (SD: 3.5). Five themes emerged: (a) the diagnostic process; (b) the relationship with health professionals during the search for a diagnosis; (c) the world of disability; (d) living day to day; (e) the disease progression.

Narratives that justify and help confirm the traceability of the results [29] are shown in Tables 3–7.

**Table 3.** Narratives of theme 1: the diagnostic process.

Theme 1: The diagnostic process
*Recognizing early symptoms*Knowing that something is going on: *“since she was born, we knew that something was wrong, because of her behavior, since she was born, since she was very young, it wasn’t normal, we knew that something wasn’t right.”* (P6, CDKL5). *“From the moment she was born, when she was admitted, from the moment she started having seizures a few hours after she was born, the world came crashing down on us. We knew there was something serious, because otherwise you don’t have to change hospitals.”* (P1, KCNQ2). Health professionals downplay importance: *“She was born with a tic in her eye, I saw that this wasn’t normal, but I took her to the pediatrician, and they didn’t give it any further importance; at two months she started having very strange movements that aren’t normal, they said it was colic. I decided to record it with my mobile phone one day, and the pediatrician sent me to the hospital emergency room, where she had her first seizure.”* (P16, GNAO1). Not wanting to see it: *“the first place where they realized that something was wrong was in the nursery. Up to that point, I don’t think we wanted to realize how far behind she was.”* (P7, CDKL5).
*The search for a diagnosis*A slow and tortuous road: *“It was a tortuous road, because of course, you get to the hospital, and they tell you that they don’t know what it could be, but they don’t send you any tests to find out, and you go around from one place to another.”* (P13, PCDH19). A constant struggle: *“the neurologist at the hospital here, she didn’t pay any attention to us… she said it was an infant epilepsy and that was it, she didn’t order any tests. And then I started my search for private doctors, and I went all over Spain looking for private doctors. It was an exhausting struggle.”* (P22, Dravet). Lack of knowledge of DEEs: *“… The specialist was a bit lost; we were in the hospital for two weeks and she didn’t know what it was or what tests to send him. She said she didn’t know what could be wrong.”* (P24, Dravet). Seeking alternative physicians and information: *“I started to investigate, I started to go on the internet, to search for the girl’s symptoms, the type of seizures she had, how they manifested and I found Dravet syndrome… when I found Dravet I said it has all the chances to be this, but at the same time you don’t want to believe it, because you read what it means for her life and you don’t want her to have it. We took it to the neurologist, and we had to fight a lot to ask for the genetic study.”* (P22, Dravet). Seeking help from private medicine: *“The delay and the refusal to do genetic testing was unacceptable, it was very hard to see the delay of everything. In the end we decided to go to private medicine to ask for the genetic tests we needed, even if we had to pay for everything ourselves.”* (P13, PCDH19).
*Receiving the diagnosis*A devastating diagnosis: *“What shocked me the most in the whole diagnosis was that when we were given the diagnosis we were told: “your child has this, and your child won’t do anything in the future”. So that is hard, that is very hard.”* (P9, CDKL5). Attitude towards the diagnosis: *“We heard from a professional that we were bringing little more than a corpse to the consultation when we went there. So, when they tell you that you have two options, either to react positively or to die, and I chose the first option, in other words, we have to fight and we have to get this baby out of here.”* (P10, CDKL5). Usefulness of the diagnosis: *“For many years, I didn’t want to put a label on it because I was afraid. Once I found it, it helped me to place him in the world and to know what to expect, but this label is also full of uncertainties.”* (P27, GNAO1).

**Table 4.** Narratives of theme 2: relationship with health professionals during the search for a diagnosis.

Theme 2: Relationship with health professionals during the search for a diagnosis
*A matter of luck*When the pediatrician downplays the importance of the illness: *“We took her many times to the pediatrician, but the pediatrician never listened to us, he said that she had a delay and that it could be normal. We went to the pediatrician many times, a number of times, but he always said no.”* (P6, CDKL5). The process depends on the neurologist: *“As soon as the lab tests arrived, the neurologist came and told us about it, but this lady had less skills to connect somewhat with parents. We requested a change of neurologist and with him, we felt much closer, he explained it to us and it calmed us down a little bit. The experience depends a lot on which doctor you get.”* (P12, GNAO1). Treatment by the neurologist: *“this can be a torture, anguish, being able to see the neurologist gives us peace of mind. In addition, she gave us the reassurance of saying things little by little, that each child must be evaluated individually. And all this helped us a lot.”* (P3, KCNQ2).
*Communicating the diagnosis*The importance of how it is communicated: *“You can’t talk like that to a family to whom you are giving a diagnosis like Dravet Syndrome and just blurt it out like that… it tears you apart, it tears you apart. Of course, I stopped listening, when he told me yes, I just cried, I just cried, I just cried, I just cried.”* (P21, Dravet). Saying it point-blank: *“The neurologist was no softie, that is, as soon as she saw what it was, she didn’t soften it. She blurted out point-blank that it was KCNQ2… but I guess there’s no better way to do it.”* (P3, KCNQ2)*.*Progressively prepare for the diagnosis: *“The diagnosis did not come out of the blue, but the neurologist did it very well. First, she opened the possibility that it could be Dravet syndrome, then we were told that she had compatible symptoms. So, it went progressively, little by little.”* (P26, Dravet). Providing too much information: *“Some doctors bombard you with information without moderation and there comes a time when you must stop them. So, when in the same conversation you are told 15 times “very poor prognosis”, you say: “I already heard you the first, the second and the fifteenth time”, because in the end you become overwhelmed. So, I think that in that case it was a punch in the face of a medical social failure, in the face of a failure of verbal communication by the doctor towards us.”* (P2, KCNQ2).

**Table 5.** Narratives of theme 3: the world of disability.

Theme 3: The world of disability
*Changing roles: assuming the role of a caregiver*Letting go of the role as a partner: *“There comes a time when you are so focused on the child that you stop being you, you stop being a woman with a husband and you start taking care of your daughter. And that’s how your 24 hours a day are summed up. Always looking after her, always on alert.”* (P3, KCNQ2). Relinquishing the role as a friend: *“This absorbs you, maybe I went overboard, but I was, and I am always looking out for him. I don’t go out with friends, I don’t do anything but look after him and take care of him, but that’s what I must do as a mother, I am his mother.”* (P1, KCNQ2). Mutual dependence: *“a bond is generated, a kind of dependency… I know that he depends on me, but I also depend on him. I can’t do anything without him or without thinking about him.”* (P7, CDKL5).
*Shattered expectations of motherhood/fatherhood*Loss of expectations: *“let’s see, when you get pregnant and you are going to have a child, you have some expectations, but that happens to everybody. Everyone wants the child to be very smart, to be an engineer, to be a doctor, and of course this… is a sudden change in the expectations you have. And well, it’s sad, because you don’t expect that this is going to happen to you, and it means a change in the concepts of everything. Of having children, of how your life is going to be, how your daily life is going to be, etcetera.”* (P26, Dravet).**Not coming to terms with the disease: *“the person I love most in this life is my daughter, she is who I love the most, but if I could go back, I wouldn’t have her. Seeing her suffer every day is the worst thing that can happen to me. I believe that the suffering of a child is the worst thing that any mother can feel, but to see my daughter suffer every day of her life is something that I can’t, I can’t overcome.”* (P10, CDKL5).

**Table 6.** Narratives of theme 4: living from day to day.

**Theme 4: Living from day to day**
*Living in the present and not thinking about the future*Not making long-term plans: *“To be honest, I never make plans… thinking ahead about the future, well, I don’t… I don’t do it very much. I live mostly on a day-to-day basis because I don’t know what’s going to happen.”* (P24, Dravet). *“I don’t have any goals in my life, I have let go of them, what we do is live day by day, whatever it can offer us and that’s all”* (P10, CDKL5). Not thinking about the future: *“The truth is that in the long term we try not to think about it, because maybe it will change for the better and we will improve or maybe we won’t. So, we don’t think about it, we don’t think about the future. That way we suffer less.”* (P9, CDKL5). Reflecting on what will happen when the parent is gone: *“I was very afraid that I would die and wondering what would become of Manu. By law of life, I hope I die before my son, but of course he can’t fend for himself, so we needed a sibling so that he could have someone to be with him in the future.”* (P2, KCNQ2). A difficult decision: *“We can’t help but think that we only have two options, leave him in the care of a relative, especially his brother, who will take care of him, or put him in a chronic care facility [silence 10 seconds]… Either option is extremely hard for us”.* (P7, CDKL5).
*Learning to live with the disease*Normalizing the situation over time: *“In the stage we are in, you normalize it more. Before, when he had a low-grade fever you worried a lot, now we know that we will have more crises, more movements, rescue medication…etc., the experience counts, when you start with the first seizures, you don’t even know where to take it, now it’s part of your life, everything doesn’t seem so strange…you don’t experience things the same.”* (P17, GNAO1)*.*Adapting to situations: *“Look, I swear that right now I always go into automatic mode. If you would have asked me a few years ago I would have said it was crazy, I would have said it was horrible… but now for me it’s like I go into automatic mode, in other words, when the child has a seizure, I don’t think, I put her in the position, I see what kind of seizure it is, and I act. I have already adapted, and I have learned.”* (P22, Dravet). Learn to fight constantly: *“The experience I have now and the fighter I am now is precisely because of everything I experienced. Because of everything I have had to battle through in this process, I learned the hard way. So, I wouldn’t change anything, because to change anything would be to not have my daughter. And the truth is that having her already makes everything worth it. It isn’t an ideal life, it’s not the motherhood I had dreamed of, nor idealized. But it’s the one I was given, and I wouldn’t change anything just for her, because to change anything would mean not having her, I just have to fight for her.”* (P3, KCNQ2).

**Table 7.** Narratives of theme 5: progression of the disease.

Theme 5: Progression of the disease
*Uncertainty and fear of disease progression*Uncertainty: *“For us, the progression is an uncertainty now, we don’t know it and we don’t know what will happen with it. You cannot have other cases as a reference because they are very different even though they have the same mutation. Each child is different, even if you have many things in common, it’s not the same.”* (P21, GNAO1). Fear: *“assuming the fear, knowing that there could be a seizure at any moment and wondering how the disease is going to develop and evolve. He has already gone through the most delicate moments, but then the teenage years come, there is a part of the future in which possible mental disorders are also associated and that’s frightening.”* (P12, PCDH19). As the disease progresses, expectations are reduced: *“managing to get the seizures to go away, to sleep better at night, to be more active during the day and to make much more progress for her to continue in an inclusive school, so that she doesn’t have to go to a special school. That’s the great hope I have. With that, I am satisfied.”* (P10, CDKL5).
*Weighing the good against the bad*Valuing the good and bad: *“It’s hard to face it, with resignation. You have no other choice, it’s a very screwed up life, life stops at the drop of a hat. But on the other hand, she gives us a lot of good things, she is a cheerful girl who brings many things to our lives. Let the bad times pass as soon as possible. I see myself in the obligation as a mother and as a person to be happy and to make my family happy, especially my daughter.”* (P19, GNAO1). The child’s happiness: *“He’s happy. I’m sure, I have no doubt. The rest of us are the ones who suffer when we see the difficulties. He doesn’t. He lives in his world where he is happy. I am sure of that because he shows it with his smile, his laughter, his hugs…”* (P1, KCNQ2).

### The diagnostic process

#### Recognizing early symptoms

With the first seizures, many parents were confronted with the appearance of multiple and changing symptoms. Participants recounted how, early on, they knew something was wrong with their child’s development. They observed different behaviors and cognitive difficulties that raised suspicions such as lack of response to parental stimuli. Becoming aware and accepting the presence of problems in their children provoked feelings of anguish and suffering due to the uncertainty of what was going to happen. Many parents reported that, upon detecting symptoms, they visited the health services, and the professionals downplayed the importance of the symptoms.

In addition, many participants reported that they avoided “seeing the symptoms”, assuming that it was just a benign epileptic seizure or an infant seizure. In many cases, this failure to see what was happening lasted until the day care center raised the alarm due to the presence of motor and/or cognitive developmental delay. Some participants explained that not being able/wanting to “see” what was happening could have been related to the fact that they were first-time parents.

#### The search for a diagnosis

The parents described the diagnostic process as a tortuous path in which they felt alone and experienced great uncertainty and uneasiness. There is no average time frame for the diagnostic process until the participants obtain the genetic tests to confirm the diagnosis. However, regardless of the time elapsed, parents experienced this phase with great distress, perceiving that they were fighting against the health system and professionals to be able to perform the diagnostic tests. Some parents believed that this struggle stemmed from the lack of knowledge about DEEs in society and the health system. When they failed to obtain a response from their referral hospital for genetic testing, they sought alternatives such as visiting private physicians, to justify and demand the genetic tests required for the diagnosis.

#### Receiving the diagnosis

Parents of children with DEE recounted that the moment they received the diagnosis was shattering, the “pandora’s box was opened” and at that moment they lost the little hope they had left. Some parents narrated how, prior to the delivery, the professionals prepared them to accept the possibility of illnesses appearing in their child. Thus, when they received the diagnosis, the shock was somewhat lessened.

The participants recounted how the diagnosis changed their whole lives, “their world fell apart”, having to face a disease for which there was no cure. Some parents decided to fight and accept the news in order to move forward. On other occasions, parents did not come to terms with the diagnosis because of its harshness. However, most of the participants agreed on the usefulness of giving a name to what was wrong with their child, and thus being able to direct the treatments. The diagnosis triggered fear, but also a certain tranquility, by reducing uncertainty and knowing what the problem was.

### Relationship with health professionals during the search for a diagnosis

#### A matter of luck

Parents described how, within the public health system, coming across a good professional was a matter of luck. If they got lucky it meant that they came across a professional who believed the parents and the problems they identified in their child, and therefore the reason for seeking help was justified. In our study, after detecting the first symptoms, parents of children with Dravet and CDLK5 went to the pediatrician and felt unlucky because the professional downplayed the importance of their demands. The diagnostic process is influenced by the medical specialist in charge, therefore certain professionals order genetic tests quickly, shortening diagnostic times and improving treatment and communication with the parents. The parents also emphasized the role of neurologists/neuropediatricians in the diagnosis.

#### Communicating the diagnosis

The manner the diagnosis was communicated by the health professional is a crucial aspect of how care is perceived by the parents. Two different approaches are described; (a) a progressive preparation by the physician, for the moment of diagnosis, giving time to assume different options little by little; and (b) the “point-blank” approach, without preparation, which provoked shock to all parents. In addition, many professionals took advantage of the moment of communicating the diagnosis to provide further information to the parents, bombarding them with excessive information, and causing the opposite effect: a greater sense of anxiety and anguish.

### The world of disability

#### Changing roles: assuming the role of a caregiver

Our participants described the loss of roles as they began caring for their children and entering “the world” of disability. They progressively stopped performing their roles as a couple, socially and at work, to exclusively devote themselves to the role of caregiver. Parents pointed out the impact on life as a couple due to the high demand for care or the need to be always on alert for seizures. In their social relationships, they stopped going out with friends because most of the time they were taking care of the children. As a result, many participants described how a bond of mutual dependence was created between them and the children.

#### Shattered expectations of motherhood/fatherhood

The expectations about parenthood were shattered with the arrival of the first crisis and, subsequently, with the confirmation of the diagnosis. The parents narrated how the loss of expectations meant a change in their lives, accompanied by a great sadness. Three participants with children with KCNQ2, Dravet and CDKL5 indicated that they love their children, but were unable to accept the disease because of the severity of the symptoms and their suffering. At times, this led to feelings of rejection of their children, mixed with grief and sadness.

### Living from day to day

#### Living in the present and not dwelling on the future

The parents live from day to day, trying not to make plans for the future. The daily difficulties prevent them from making medium or long-term plans, because seizures could appear at any time, and disrupt any anticipated plan. Parents must live in the moment and plan “on the fly”. In addition, parents described worrying about the future, when they would grow old or die. Their children were growing up, making it more difficult to care for them (their strength and size increased) and to manage their symptoms. They described how the options they considered included institutionalization in specialized centers, or leaving them in the care of relatives, especially siblings.

#### Learning to live with the disease

Over time, the participants normalized their lives and were able to learn “as they went along”. The parents pointed out how the people around them did not understand this “normalization” of the disease and the daily care and management. Nonetheless, within the constant struggle that the parents assumed from the onset of the disease, they acknowledged that there is a certain learning process, which allows them to be more capable in the care and management of symptoms and helps them to grow.

### Progression of the disease

#### Uncertainty and fear of disease progression

A key issue acknowledged by parents is the uncertainty regarding the progression of the disease. The variability in the presentation of symptoms, even within a single variant of DEE, is a stressor in the course of the disease. Parents of children with DEE reported that the absence of a clear prognosis in the evolution of the disease caused them great uncertainty, which was accompanied by fear of not knowing whether symptoms would increase in adolescence, or whether sequelae derived from epileptic seizures would appear, that can lead to disability and dependence. In addition, parents described how their expectations of improvement and/or a cure decrease as the years go by, evolving into their main wish, which is for their children to be happy or to have a good quality of life, regardless of their limitations.

#### Weighing the good against the bad

The parents have endured very hard times, with a significant emotional burden, accompanied by great fear and uncertainty, having to witness the suffering of their children. Nevertheless, despite the suffering, over time, they have been able to perceive and appreciate the moments of happiness they experience with their children and to value other aspects of life such as companionship, affection and the bond generated in the face of adversity between the members of the couple and the family.

## Discussion

Our results show the impact that DEEs have on the parents in their daily lives in relation to diagnosis, disease management and disability.

### The diagnostic process

DEEs are characterized by early onset seizures and numerous comorbidities [12,33]. However, there is much heterogeneity regarding the clinical manifestations of DEEs [9]. The difficulty of recognizing symptoms in the first months of life is common in many DEE due to the heterogeneity of manifestations and lack of information [34–36]. Palacios-Ceña et al. [34] described how the parents of children with Rett syndrome did not perceive the symptoms, even though the functional impairment was evident. In contrast to our results, in the study by Palacios-Ceña et al. [34], it was the parents, not the teachers, who raised the alarm and went to the health professionals.

The diagnosis of a DEE is described as an extensive process, where parents “struggle” to receive the diagnostic tests their children need [37–39]. Previous studies [37,40] showed how DEEs are difficult to diagnose due to lack of knowledge regarding the most appropriate tests for each disease, and the heterogeneity and/or variability of clinical manifestations, leading to repeated testing, diagnostic delay, and, consequently, delayed treatment. Jeffrey et al. [41] described the diagnostic process of a DEE as “a journey” in which the prescription of specific tests is delayed. In the case of Dravet syndrome, Juandó-Prats et al. [5] noted how most children are diagnosed before the age of one year, as seizures usually appear within a few months of birth. However, this is variable among DEEs. According to our findings, parents did not recall the mean time it took to reach the diagnosis, nonetheless, they described it as a distressing process, irrespective of time. Köstner et al. [42] pointed out the importance of early diagnosis of epilepsy to minimize the damage and begin appropriate treatment. Our results showed how the diagnostic delays force parents to seek consultations in the private health system including access to diagnostic tests.

Jeffrey et al. [41] pointed out how the genetic diagnosis of a DEE helped the parents to know the trajectory of the disease and to establish a perspective regarding the future. These authors [41] pointed out that upon diagnosis, a psychological turning point appears, which could help them to mitigate family stress and trauma. Previous studies [38,41] described how receiving a correct diagnosis and putting a “name” to their child’s situation was considered of great importance to parents of children with DEE. Similarly, other research [38] has described how putting “a label” to the diagnosis helps parents understand DEE such as Phelan McDermid, as well as gaining greater understanding of their children’s behaviors, and thus targeting their efforts towards effective treatments. García-Bravo et al. [38] showed that with the establishment of the diagnosis, parents’ feelings of guilt, uncertainty and/or pain lessened.

### Relationship with health professionals during the search for a diagnosis

Tschamper et al. [43] described how parents of children with epilepsy suffered distress when treated by professionals who were unfamiliar with this type of disease, and the emotional burden increased when the epilepsy was severe. In a study by Juandó-Prats et al. [5], among parents of children with Dravet, parents highly appreciated that the medical specialists valued aspects of the entire family’s well-being, as this was a point that was not usually considered by the professionals. Our results showed how parents positively viewed the professionals’ “sensitivity” to their needs and demands, which was perceived as “being lucky” because it was not what they expected from their previous experiences with other professionals during their child’s diagnosis. Previous studies [6,34] among children with DEE reveal that parents sometimes feel alone in the search for solutions and tests for their children, as their concerns are not considered by health professionals and/or are minimized.

Other studies [18,38,44] highlight that the communication of the diagnosis to parents is a key moment for facilitating understanding and coping with the disease and parental involvement. In addition, Germeni et al. [44] distinguish between deficient and effective communication, based on the involvement and interest of the health professional. Our results show the relevance given by parents to the communication of the diagnosis, and how preparation with the help of specialists can reduce the shock of the moment of diagnosis.

Tschamper et al. [43] in their work with parents of children with epilepsy, described how at the time of diagnosis, health professionals were not able to provide them with useful information about the specific DEE of their children, only receiving information about epilepsy in general. In contrast, our results show how professionals took advantage of the diagnosis to provide more information, which had the opposite effect on the parents, making them feel overwhelmed or confused.

### The world of disability

Previous studies [5,13,45] have demonstrated how the life of the parents and their family is altered by the presence of epileptic seizures, highlighting the continuous care needs, and the burden of the disease and its impact, all of which are aggravated in the case of DEEs. Van Westrhenen et al. [46] described how parents of children with epilepsy developed overprotective behaviors towards their children, which affected the overload experienced by the parents. This was exacerbated during nighttime vigilance, directly affecting the parents’ rest and sleep. Moreover, the role of caregiver in parents of children with DEE places a burden on parents’ mental health, modifying their participation and restricting their work, social or family life due to the high demand for care and responsibilities of children with DEE [10,47,48].

The diagnosis and impact of a disease with disabilities shatters all the meanings and expectations that parents had created about motherhood/parenthood, their child, the family, and their future [35,49,50]. Nayeri et al. [50] and Geuze & Goossensen [49] revealed how the meaning of parenthood, the cultural and social expectations linked to the meaning of what a family is, are broken and accompanied by anguish in the face of the diagnosis and daily difficulties.

### Living from day to day

In the case of Dravet, Juandó-Prats et al. [5] noted that, as seizures were one of the symptoms that parents were most concerned about, their attention was focused on the present, as their goal was to stop the progression of seizures, and their impact on the lives of all family members. The future and plans take a back seat.

Fayed et al. [11] described how parents of children with epilepsy must adapt to the disease, coping with daily challenges and daily uncertainty, waiting for the arrival of an unexpected seizure. In addition, Reeder et al. [51] described how people with epilepsy and their families feel that their lives are at a standstill waiting for a seizure to arrive. Families tend to normalize the care and management of DEEs, however, according to Clifford et al. [52], the perceived misunderstanding of families of children with epilepsy and their families contributes to a greater burden of the disease on a day-to-day basis.

### Disease progression

A factor of stress and concern for parents of children with RD is the evolution of the disease and planning for their child’s future [36,53]. Cardão et al. [53] and García-Bravo et al. [36] described the presence of overwhelming fear in parents of children with RD when thinking about the future, accompanied by stress, anxiety, sadness and/or grief.

Our results show how parents balance the negative and positive moments of caring for their child with DEE. However, Gallop et al. [12] in their literature review on the impact of DEEs on caregivers, noted that most of the available studies highlighted the negative impact on parents of caring for children with DEEs, with no reference to the positive aspects in most cases. However, according to previous studies [45,54] on DEE, although parents described how day-to-day difficulties lead to feelings of stress, anger, mood swings or somatic symptoms, they also recognized how the disease produced stronger family cohesion, facilitated the vision of new life perspectives, greater personal strength or the acquisition of new skills that make them better caregivers [45,54]. The presence of happiness and suffering are feelings referred to by parents of children with DEE, where they describe how diseases “teach them to see life differently” [33]. Previous research [36,45,54] notes that family resilience is developed after diagnosis, with parents reevaluating important aspects of life, valuing moments of joy and reinforcing participation in family routines and rituals [54].

#### Limitations

This study has limitations in terms of generalizability, i.e. the results of a qualitative study cannot be extrapolated to all parents with children with DEE. Because the study was conducted with participants outside the clinical setting, unfortunately, data and clinical reports confirming some data such as dates of medical visits and date of diagnosis could not be obtained and therefore could not be considered.

## Conclusions

Our results show the process of the diagnosis of DEE from the detection of the first symptoms by parents to the confirmation and communication of the diagnosis, along with the emergence of the caregiver’s role in the face of the child’s disability, and the need to learn to live with the disease and cope with the daily uncertainty in caring for children with DEE. These results will help healthcare professionals to support parents and improve the process of genetic and syndromic diagnosis. There is a need to develop programs that integrate health and social interventions that help parents of children with DEE in the daily care and reduce the impact of the disease on the family.

**Figure 1.**
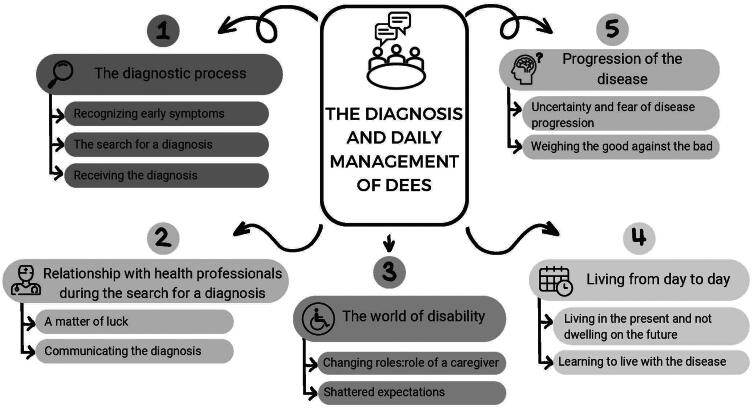
Five themes emerged from narratives.

## Supplementary Material

graphical abstract.png

## Data Availability

The data that support the findings of the study are available on request from the corresponding author, upon reasonable request. The data are not public due to ethical restrictions.
