# Supplementary figures and images for: Real-world experience of diagnosis, disability, and daily management in parents of children with different genetic developmental and epileptic encephalopathies: a qualitative study
Source: Ann Med. 2024 Dec 28;57(1):2446702. doi: 10.1080/07853890.2024.2446702 (PMC11703127; doi:10.1080/07853890.2024.2446702)

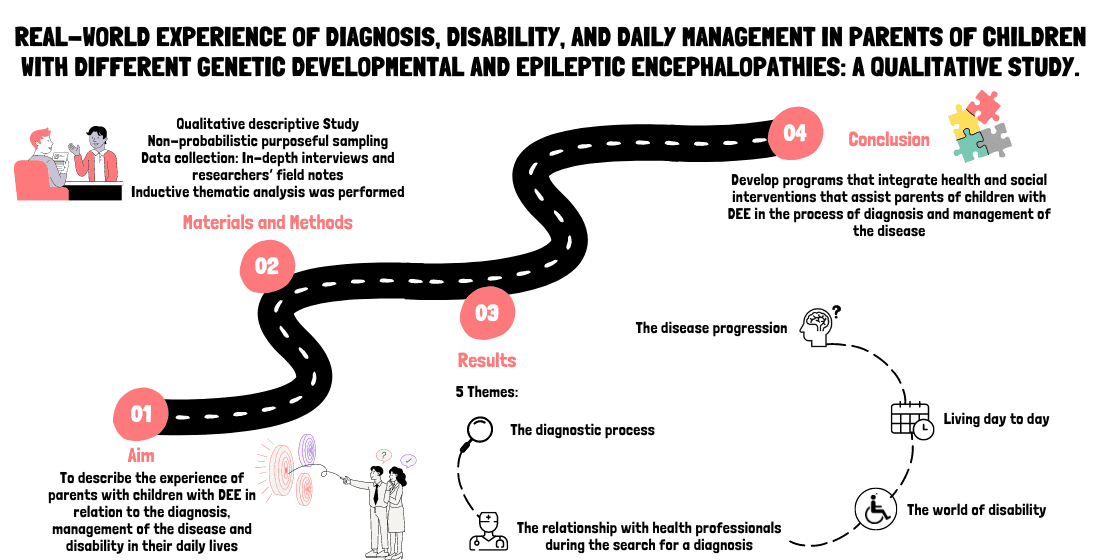

Supplement: graphical abstract.png [file IANN_A_2446702_SM3556.png]
